# Effect of a mobile digital intervention to enhance physical activity in individuals with metabolic disorders on voiding patterns measured by 24-h voided volume monitoring system: Kumejima Digital Health Project (KDHP)

**DOI:** 10.1007/s11255-021-02867-x

**Published:** 2021-04-28

**Authors:** Minoru Miyazato, Asuka Ashikari, Koshi Nakamura, Takehiro Nakamura, Kiyoto Yamashiro, Tsugumi Uema, Moriyuki Uehara, Hiroaki Masuzaki, Seiichi Saito, Shiro Maeda, Hajime Ishida, Masayuki Matsushita

**Affiliations:** 1 Department of Systems Physiology, Graduate School of Medicine, University of the Ryukyus, 207 Uehara, Nishihara, Okinawa 903-0215 Japan; 2 Department of Urology, Graduate School of Medicine, University of the Ryukyus, Okinawa, Japan; 3 Department of Public Health and Hygiene, Graduate School of Medicine, University of the Ryukyus, Okinawa, Japan; 4 Division of Endocrinology, Diabetes and Metabolism, Hematology, Rheumatology (Second Department of Internal Medicine), Graduate School of Medicine, University of the Ryukyus, Okinawa, Japan; 5 Department of Advanced Genomic and Laboratory Medicine, Graduate School of Medicine, University of the Ryukyus, Okinawa, Japan; 6 Division of Clinical Laboratory and Blood Transfusion, University of the Ryukyus Hospital, Okinawa, Japan; 7 Department of Human Biology and Anatomy, Graduate School of Medicine, University of the Ryukyus, Okinawa, Japan; 8 Department of Molecular and Cellular Physiology, Graduate School of Medicine, University of the Ryukyus, Okinawa, Japan

**Keywords:** Lifestyle, Metabolic diseases, Nocturia, Polyuria, Voided volume

## Abstract

**Purpose:**

To evaluate the effect of a mobile digital intervention on voiding patterns, we performed 24-h voided volume monitoring in individuals with metabolic disorders.

**Methods:**

Participants with metabolic disorders were grouped into either the intervention group (*n* = 17), who had access to a smartphone app (CARADA), or the non-intervention group (*n* = 11), who did not. Urine monitoring was conducted for 24 h using a novel digital self-health monitoring system for urine excretion (s-HMSU). Body weight, abdominal circumference, blood pressure, and biomarkers were measured.

**Results:**

Physical findings and blood test results at baseline and 6 months indicated no significant between-group differences. Night-time frequency did not change between baseline and 6 months in the intervention group but significantly worsened at 6 months in the non-intervention group, as compared to baseline (1.0 ± 0.7 vs. 1.5 ± 0.5, *p* < 0.05). The change in night-time frequency over 6 months did not differ between the intervention and non-intervention groups. Furthermore, the change in hours of undisturbed sleep over 6 months did not differ between the two groups. However, compared with baseline, nocturnal polyuria index tended to worsen at 6 months in the non-intervention group.

**Conclusion:**

Our study results suggest that mobile digital intervention might be useful for behavioral therapy to improve night-time frequency and urine production and that s-HMSU might be beneficial for confirming the prevention of progress in individuals with metabolic disorders, which can aid in modifying lifestyle.

## Introduction

Voiding behavior is a daily activity; therefore, voiding dysfunction significantly deteriorates health-related quality of life [1]. Nocturia, in particular, disturbs sleep, increases the risk of falls and hip fractures at night, and affects mortality rate [2]. The etiology of nocturia is multifactorial, and nocturia has been reported to be closely related to metabolic syndrome [3, 4]. These facts are mostly based on various community-based epidemiological studies [2–4]. Nonetheless, whether nocturia can be a surrogate marker of metabolic syndrome remains unknown. To better understand the association between nocturia and metabolic syndrome, a longitudinal study or an intervention study conducted on individuals with nocturia is necessary. However, assessment of nocturia is difficult due to its multifactorial etiology.

Nocturia originates from overproduction of urine, reduced bladder capacity, sleep disorders, or a combination of these factors; the former is reported to be the main cause, at approximately 80% of cases [5, 6]. Frequency-volume charts are mandatory for the diagnosis of the underlying causes of nocturia. Traditionally, paper-based frequency-volume charts for 1–7 days have been used; however, analogue charts are time-consuming to complete, resulting in a low adherence rate [7]. The digital self-health monitoring system to measure the volume of voided urine (s-HMSU) for home use was originally developed as an alternative to paper-based frequency-volume charts. We previously validated the measured voided volume of the s-HMSU system and confirmed its high reliability in the Kumejima Digital Health Project (KDHP) [8]. Automatically collected data include night-time frequency, maximal bladder capacity, and hours of undisturbed sleep (HUS) [9]. These can be surrogate markers of metabolic syndrome. Therefore, in the present study, to confirm the feasibility of a mobile digital intervention and to look for a surrogate marker of metabolic syndrome, we performed 24-h voided volume monitoring in individuals with metabolic disorders.

## Methods

### Study design and participants

This interventional study was conducted as a part of the primary intervention study of the KDHP between June 2018 and December 2019. The primary intervention study aimed to investigate whether digital health approaches using a smartphone app (CARADA, MTI Ltd., Tokyo, Japan) with feedback for 15 months improved obesity and metabolic disorders by reinforcing lifestyle modification.

**Fig. 1:**
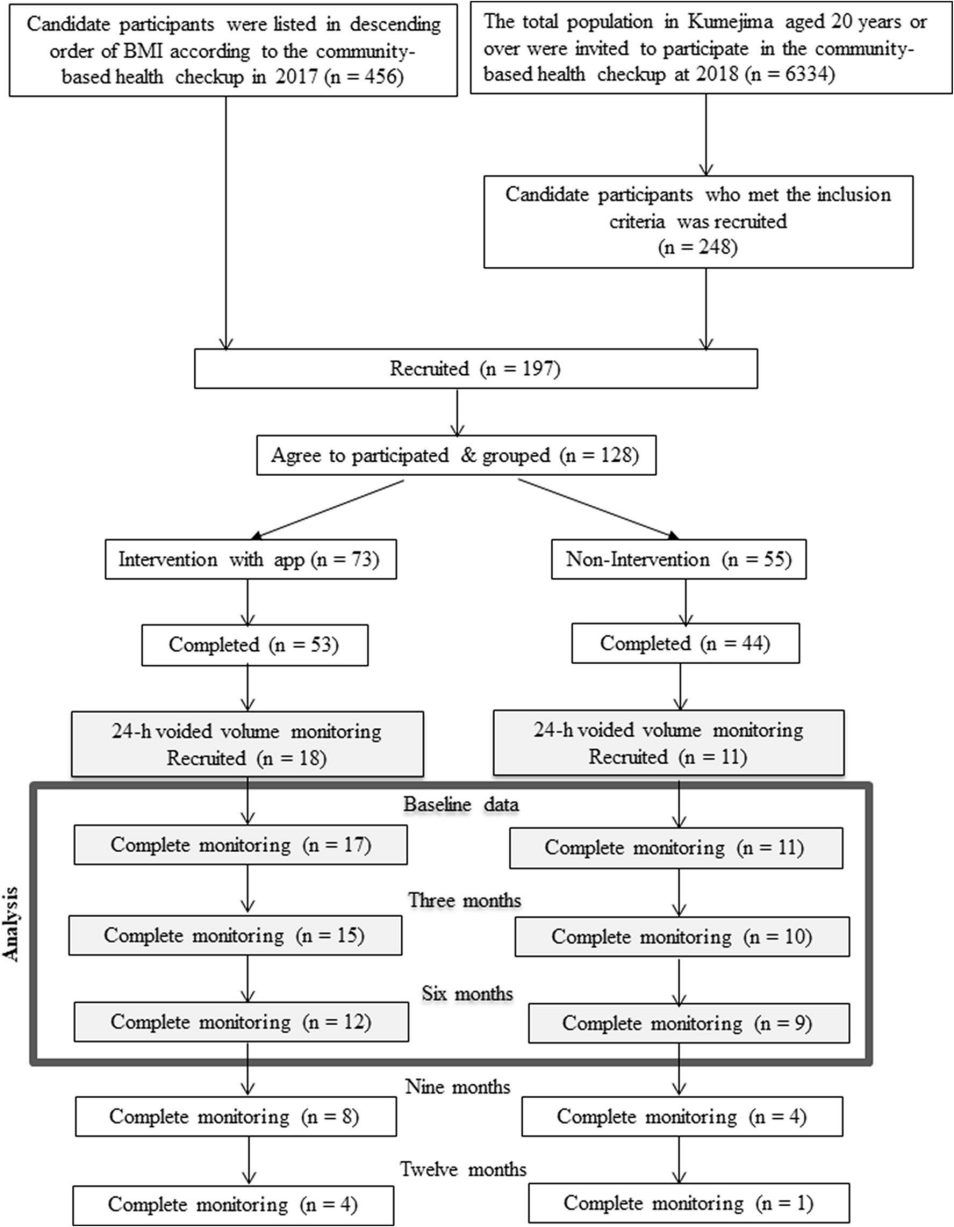
Flow of participants using 24-h voided volume monitoring. **a** Intervention group and **b** non-intervention group. BMI, Body Mass Index

Participants aged ≥ 20 years, with obesity and/or metabolic disorders, were identified based on a community-based health checkup in 2017, the year before the primary intervention of this study. A total of 456 candidates were listed in descending order of body mass index (BMI), were in advance randomly assigned to either the intervention group or the non-intervention group, and were invited to participate in this study. Furthermore, an additional 248 candidates who met the inclusion criteria were recruited at a community-based health checkup in 2018 and were grouped similarly. Of the 197 candidates (110 in the intervention group and 87 in the non-intervention group), 128 voluntarily participated in this intervention study after providing written informed consent. Of the 73 and 55 participants in the intervention and non-intervention groups, 53 and 44, respectively, completed 15-month observation (Fig. 1). The inclusion criteria were as follows: (1) obesity, defined as BMI ≥ 25 kg/m^2^ [10]; (2) metabolic syndrome, defined as abdominal circumference ≥ 85 cm for men and ≥ 90 cm for women, accompanied by any two of three disorders (high blood glucose, dyslipidemia, and high blood pressure) [11]; (3) pre-diabetic, defined as fasting blood glucose 110–125 mg/dL, occasional blood glucose 140–199 mg/dL, or blood glucose 140–199 mg/dL after 2 h on 75 g oral glucose tolerance test [12]; and (4) insulin resistance, defined as fasting insulin ≥ 15 µU/mL or index of homeostasis model assessment of insulin resistance ≥ 2.5 [13]. For the three components of metabolic syndrome, high blood glucose was defined as fasting blood glucose ≥ 100 mg/dL or use of medication for diabetes, dyslipidemia as serum triglyceride ≥ 150 mg/dL or high-density lipoprotein cholesterol < 40 mg/dL, and high blood pressure as systolic blood pressure (SBP) ≥ 130 mmHg, diastolic blood pressure (DBP) ≥ 85 mmHg, or use of medication for hypertension [11].

Of the 53 and 44 participants in the intervention and non-intervention groups, 17 and 11, respectively, were involved in the present study of additional data collection on urination. Finally, 21 participants (12 in the intervention group and 9 in the non-intervention group) who provided complete urination data at baseline and 6 months were included in the analysis. The study protocol complied with the Declaration of Helsinki and was approved by the Ethics Committee of the University of the Ryukyus for Medical and Health Research Involving Human Subjects (#1170). All participants provided written informed consent. This study was registered in a public trial registry in Japan (UMIN ID: 000032453; https://www.umin.ac.jp/).

### Intervention

The intervention group had access to a smartphone app (CARADA), on which they could record walk counts, weight, diet, and sleep automatically or by themselves. Using the self-monitoring data collected by the app, we created a new program to generate personalized health advice for the participants and delivered them regularly via email only in the intervention group. The intervention group could view their data on the app and weekly health messages on the smartphone at any time, while the non-intervention group could only view their biochemical test results.

### Urinary tract symptoms questionnaire

Participants completed a baseline questionnaire including lower urinary tract symptoms (Core Lower Urinary Tract Symptom Score questionnaire [CLSS]) and the Quick Inventory Depressive Symptomatology-Japanese (QIDS-J) [14]. Nocturia was assessed as the overall night-time frequency based on Q2 in CLSS, while storage symptom was assessed as urgency (Q3). Mid-nocturnal insomnia was also based on Q2 in QIDS-J.

### 24-h voided volume monitoring system

**Fig. 2:**
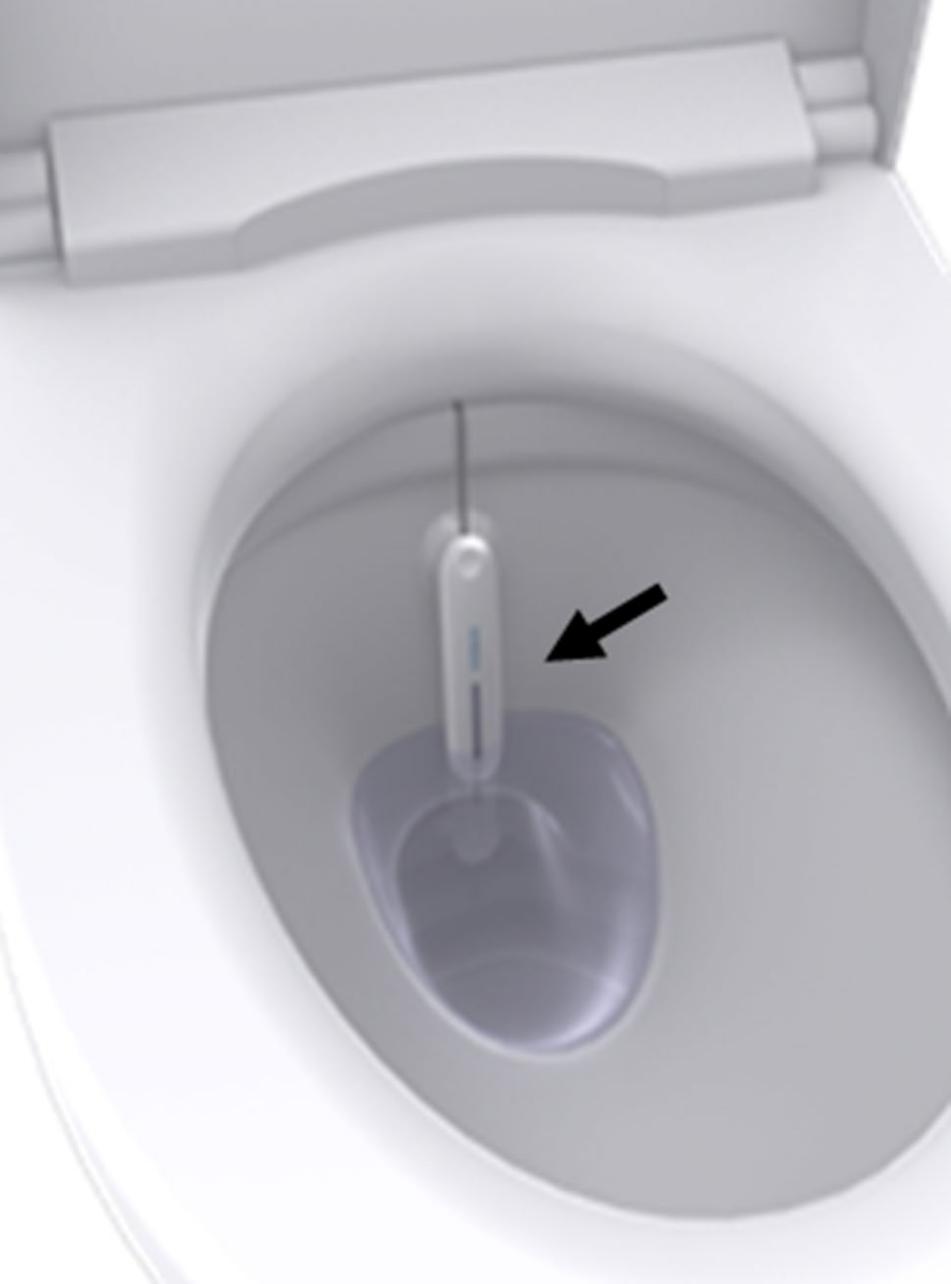
The s-HMSU system includes a server, measurement device, and a user terminal. The sensor is located in the water in the toilet bowl (black arrow). s-HMSU, self-health monitoring system for urine excretion

To measure 24-h voided volume, we used the s-HMSU system (Symax Inc., Tokyo, Japan). The s-HMSU system includes a server, a measurement sensor (Fig. 2), and a user terminal. The sensor is installed on the toilet itself and calculates the volume of urine excreted using a fluid model, including the shape of the toilet bowl and the known volume and temperature of the water in the bowl. We have previously validated the measured voided volume of the s-HMSU system and confirmed reliable and high resolutions (intraclass correlation 0.972) [8]. Each participant could be recognized by an identification card, and the voiding volume of excreted urine for each participant was automatically recorded. We equipped the home toilets of individuals with the s-HMSU. Calibration was performed by calculating the coefficient, which depends on the shape of each toilet bowl and the known volume and temperature of the water in the bowl. The recorded data also included the start and end times of voiding.

Automatically collected data included daytime urinary frequency, night-time frequency, daytime urine volume (mL), nocturnal urine volume (mL), maximal bladder capacity (mL), and HUS (min). HUS was defined as the time from falling asleep to first awakening to void [9]. Nocturnal polyuria index (%) was calculated as (nocturnal urine volume (mL)/ daytime urine volume (mL) + nocturnal urine volume (mL)] × 100.

### 24-h voided volume monitoring schedule

The participants in both the intervention and non-intervention groups continued with their usual care. Due to time to examine each home toilet type and to preparation, 24-h voided volume monitoring was started 2–12 months after starting the application in the intervention group (Table 1). Each participant was monitored for 24 h at baseline and at 3, 6, 9, and 12 months. The data regarding time the participant woke or went to the bed were collected by the app in the intervention group; these data were self-recorded in the non-intervention group. On the monitoring day, daily water intake was also recorded in both groups.

**Table 1:** Baseline characteristics (*n* = 28)

Characteristics	Non-intervention (*N* = 11)	Intervention (*N* = 17)	*p* value
Age (mean years, SD)	55.8 (12.8)	49.2 (12.8)	0.13
Gender (male:female)	8:3	12:5	0.90
BMI (SD)	27.8 (3.0)	28.2 (3.9)	0.69
Night-time frequency (cm, SD)^a^	0.88 (0.78)	0.73 (0.65)	0.65
Urgency (median, interquartile range)^a^	0 (0–1)	0 (0–1)	0.71
*Education, no (%)*			
High school graduate or less	6 (54.5%)	7 (41.2%)	0.49
Above high school graduate	5 (45.5%)	10 (58.8%)	
Abdominal circumference (cm, SD)	96.8 (6.5)	95.1 (9.8)	0.23
SBP (mmHg, SD)	139.9 (11.5)	133.1 (11.8)	0.18
DBP (mmHg, SD)	86.5 (10.7)	83.0 (11.0)	0.54
Creatinine (mg/dL, SD)	0.75 (0.16)	0.84 (0.20)	0.13
Fasting glucose (mg/dL, SD)	89.3 (11.7)	84.3 (7.1)	0.44
HbA1c (%, SD)	5.8 (0.5)	5.6 (0.3)	0.41
Triglyceride (mg/dL, median, interquartile range	129 (100–328)	121 (88.5–189.5)	0.50
Total cholesterol (mg/dL, SD)	219.3 (31.9)	214.4 (28.4)	0.78
Time at starting 24-h urine volume monitoring from intervention (month, median, interquartile range)	6 (4–8)	5 (4.5–9.5)	0.85

Analysis by Wilcoxon signed-rank test

BMI, Body Mass Index; SBP, systolic blood pressure; DBP, diastolic blood pressure; HbA1C, hemoglobin A1C; SD, standard deviation

^a^Based on Core Lower Urinary Tract Symptom Score questionnaire

### Additional data collection

Fasting blood samples were obtained, and glucose, hemoglobin A1c (HbA1c), serum creatinine, triglyceride, and total cholesterol levels were measured using standard procedures. Blood pressure was measured in the sitting position once on the right arm using a standardized device. Height, weight, and abdominal circumference were measured, and BMI was calculated as weight in kilograms divided by the square of height in meters (kg/m^2^). These variables were examined every 3 months for 15 months. History of education was evaluated by a self-administered questionnaire.

### Outcomes

The primary outcome was a change in night-time frequency for 6 months. A decrease in night-time frequency indicated an improvement in nocturia. The secondary outcome was change in HUS, nocturnal polyuria index, or maximal bladder capacity for 6 months. An increase in HUS and maximal bladder capacity and a decrease in nocturnal polyuria index indicated improvement of each parameter.

### Statistical analysis

Data are expressed as mean ± standard deviation or median (interquartile range). Initially, differences between groups were examined using a non-parametric Wilcoxon signed-rank test or chi-square test. Next, we assessed whether each parameter had changed significantly over 3–6 months in the intervention and non-intervention groups using paired t tests. Then, we compared the change in night-time frequency over 6 months between the intervention group and the non-intervention group. The flow of study participants’ involvement (Fig. 1) no longer guaranteed random assignment for the intervention group and the non-intervention group. Therefore, given the confounding effects between these two groups, we compared the change of interest, using an analysis of covariance that incorporated the following variables as covariates: age, sex, baseline night-time frequency, time at starting 24-h voided volume monitoring from enrollment, and education (high school graduate or less, and above high school graduate). We have previously observed significant differences in maximum bladder capacity and HUS between individuals with and without metabolic disorders in an observational study of the KDHP (data not shown); thus, the change in max bladder capacity (mL) and HUS (min) between the intervention group and the non-intervention group at 6 months was also analyzed. The main analysis was repeated after study participants were stratified by sex. All statistical analyses were performed using JMP version 9.0 (SAS Institute Inc., Cary, NC, USA). All probability values were two-tailed, and a *p* value of < 0.05 was considered statistically significant.

## Results

### Characteristics of the study participants

The intervention and non-intervention groups included 17 and 11 participants with obesity and/or metabolic disorders, respectively. The intervention and non-intervention groups had mean night-time frequency of 0.88 and 0.73 (more than once 71.4% in total participants), mean ages of 49.2 and 55.8 years, and a sex ratio (male: female) of 12:5 and 8:3, respectively. Median urgency was 0 in both groups in CLSS. However, 11 out of 28 participants (39.3%) felt urgency often or sometimes (score 1 or 2) (Table 1). Physical findings and blood test results at baseline indicated no significant differences between the groups.

### Urinary endpoints

#### Primary outcome

**Fig. 3:**
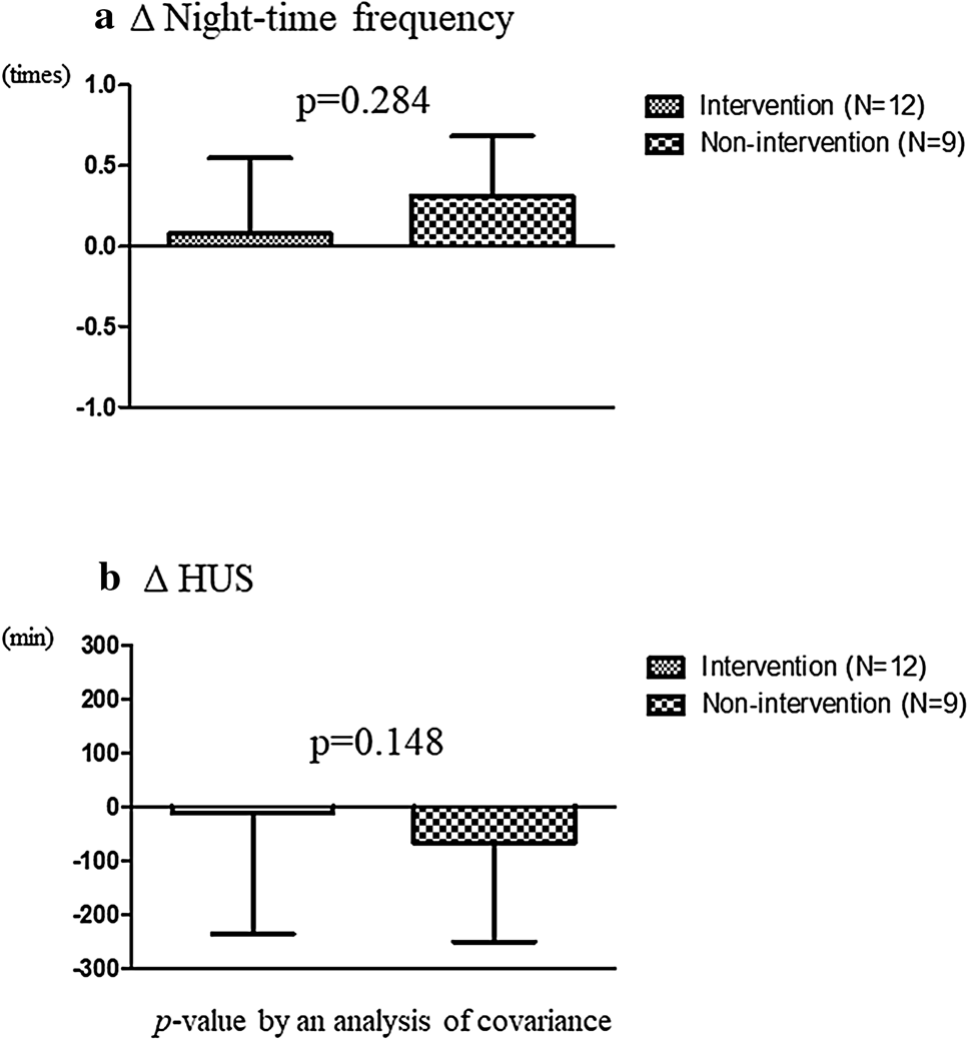
The change in night-time frequency (**a**) and HUS (**b**) in the intervention and non-intervention groups at 6 months. Analysis of covariance was used after adjustment for age, sex, baseline night-time frequency, time from enrollment to starting 24-h voided volume monitoring, and education

Night-time frequency did not change in the intervention group but significantly worsened at 6 months in the non-intervention group, as compared to baseline (1.0 ± 0.7 vs. 1.5 ± 0.5, *p* < 0.05; Table 2). The change in night-time frequency over 6 months did not differ between the intervention and non-intervention groups (*p* = 0.284; Fig. 3a). In females, the change in night-time frequency over 6 months was significantly larger (*p* = 0.049) in the intervention group compared with the non-intervention group. However, night-time frequency did not show significant differences in male participants between the two groups.

**Table 2:** Variables at baseline and 6 months in the intervention and non-intervention groups

Characteristics	Baseline		Six months	
	Non-intervention (*N* = 11)	Intervention (*N* = 17)	Non-intervention (*N* = 9)	Intervention (*N* = 12)
BMI (SD)	27.8 (3.0)	28.2 (3.9)	28.0 (3.5)	28.4 (4.0)
Abdominal circumference (cm, SD)	96.8 (6.5)	95.1 (9.8)	96.1 (5.9)	94.5 (10.8)
SBP (mmHg, SD)	139.9 (11.5)	133.1 (11.8)	130.7 (17.1)	133.7 (17.5)
DBP (mmHg, SD)	86.5 (10.7)	83.0 (11.0)	77.7 (13.9)	83.4 (14.7)
Creatinine (mg/dL, SD)	0.75 (0.16)	0.84 (0.20)	0.76 (0.15)	0.83 (0.21)
Fasting glucose (mg/dL, SD)	89.3 (11.7)	84.3 (7.1)	90.8 (13.5)	86.4 (6.9)
HbA1c (%, SD)	5.8 (0.5)	5.6 (0.3)	5.8 (0.5)	5.7 (0.3)
Triglyceride (mg/dL, median, interquartile range)	129 (100–328)	121 (88.5–189.5)	175 (105.5–264.5)	125 (71.5–166)
Total cholesterol (mg/dL, SD)	219.3 (31.9)	214.4 (28.4)	202.3 (24.5)	217.8 (32.0)
Daytime urinary frequency (SD)	8.1 (4.3)	5.6 (2.5)	7.0 (2.5)	6.6 (3.0)
Night-time frequency (SD)	1.0 (0.7)	1.4 (1.3)	1.5 (0.5)*	1.2 (0.8)
Daytime urinary volume (mL, SD)	590.0 (250.1)	585.5 (466.5)	593.6 (300.3)	651.3 (310.2)
Nocturnal urine volume (mL, SD)	211.7 (110.8)	226.5 (156.9)	281.9 (154.3)	243.8 (118.0)
Nocturnal polyuria index (%, SD)	30.0 (24.8)	28.6 (17.0)	34.9 (21.4)	28.3 (11.2)
Maximum bladder capacity (mL, SD)	201.0 (88.9)	228.3 (168.3)	241.5 (81.7)	232.7 (93.7)
Hours of undisturbed sleep (min, SD)	258.1 (163.8)	213.2 (177.8)	215.8 (155.3)	218.2 (148.8)
Water intake (mL, SD)	1884.1 (659.3)	1915.9 (959.5)	2512.2 (1193.6)	2203.1 (1047.6)
QIDS-J Q2 (median, interquartile range)	2 (0–3)	2 (0–2)	1 (0–2)	1 (0–2)

Analysis by paired *t* tests; *p < 0.05 vs baseline

BMI, body mass index; SBP, systolic blood pressure; DBP, diastolic blood pressure; HbA1C, hemoglobin A1C; SD, standard deviation, QIDS-J, Quick Inventory Depressive Symptomatology-Japanese

#### Secondary outcome

Compared with baseline, nocturnal polyuria index tended to worsen at 6 months in the non-intervention group (*p* = 0.0946 and *p* = 0.0833, respectively). Mid-nocturnal insomnia (Q2 in QIDS-J) did not change in either group at 6 months (Table 2). The change in HUS over 6 months did not differ between the intervention and non-intervention groups (*p* = 0.148) (Fig. 3b). The change in nocturnal polyuria index, and maximal bladder capacity over 6 months did not show any significant differences between the two groups. Physical findings and blood test results indicated no significant changes in either group.

## Discussion

The main cause of nocturia is overproduction of urine, which reaches approximately 80% [5, 6]. Nocturia has a multifactorial etiology and is closely related to metabolic syndrome [3, 4]. The primary aim of the present study was to evaluate the effect of a mobile digital intervention on voiding patterns measured by a 24-h voided volume monitoring system in individuals with obesity and/or metabolic disorders. In this study, night-time frequency did not change in the intervention group but significantly worsened at 6 months in the non-intervention group, as compared to baseline. Compared with baseline, nocturnal polyuria index (i.e., nocturnal urine volume) tended to worsen at 6 months in the non-intervention group. Hirayama et al. [15] reported that nocturnal urine volume was closely related to diurnal leg edema, suggesting that fluid retention in the leg rostrally shifts after sleep, thereby leading to the overproduction of urine at night [16]. Soda et al. [17] previously reported that behavior therapy such as walking exercise could improve nocturia by reducing the production of nocturnal urine volume. Therefore, mobile digital intervention, including reminders to perform daily exercise or avoid excessive alcohol and caffeine consumption, might be useful in behavioral therapy for nocturia. Nocturia and related symptoms such as nocturnal polyuria index may be surrogate markers of metabolic syndrome. Nevertheless, the change in night-time frequency over 6 months after adjustment for age, sex, baseline night-time frequency, time at starting 24-h voided volume monitoring, and education did not differ between the two groups (*p* = 0.284), which may be attributable to the small number of participants. In female participants, the change in night-time frequency over 6 months was larger in the intervention group compared with non-intervention group. The reason behind this sex-specific difference is unclear. Our future studies will aim to clarify this difference along with the long-term effect of an intervention. To our knowledge, this is the first prospective clinical trial to confirm that nocturia could be a surrogate marker of metabolic syndrome.

Nocturnal polyuria is unlikely to be the only component of nocturia. In a rat model of atherosclerosis-induced chronic pelvic ischemia, bladder activity was upregulated with elevated oxidative stress markers (8-hydroxy-2′-deoxyguanosine, malondialdehyde) and proinflammatory cytokines. It has also been reported that intermittent hypoxia (8 weeks) in rats induced detrusor instability accompanied by an increase in tissue malondialdehyde and oxidation-related proteins [18]. Thus, metabolic syndrome associated with atherosclerosis, especially in the pelvic artery circulating the bladder, may cause reduced bladder capacity. Indeed, 39.3% of our participants had storage symptoms as urgency (Q3 in CLSS) with relatively reduced bladder capacity (average 217.8 ml) in the present study. However, maximal bladder capacity did not change in the intervention group, suggesting due to a short intervention period of only 6 months. HUS is considered to be another key factor for nocturia-related quality of life [9]. In the present study, the change in HUS at 6 months and mid-nocturnal insomnia (Q2 in QIDS-J) at 6 months also did not differ between the intervention and non-intervention groups, suggesting due to a complicated cause of sleep disorders. On the other hand, in our recent study, tadalafil, a long-acting phosphodiesterase type 5, improved nocturia, HUS, and sleep disorders (inadequate sleep at night and overall bother) in patients with benign prostatic hyperplasia [19]. Overall, nocturia, which has a wide pathogenesis, can be useful to detect physical changes before and after intervention.

The s-HMSU was developed to provide a reliable alternative to paper-based frequency-volume charts to measure the volume of urine excretion. We have previously validated the measured voided volume of the s-HMSU system using the change in body weight and confirmed that this volume measurement was reliable (intraclass correlation = 0.972, coefficient of determination = 0.94) [8]. Automatically accumulated urine volume data provide several advantages, as individuals do not require data input and rapid transfer, such as by cloud, can make early clinical decision-making and feedback more accessible. In addition, voiding is the most basic human behavior and therefore represents daily conditions. Thus, s-HMSU was reliable and feasible for home-based automated accumulation of frequency-volume charts.

In Japan, an increase in the incidence rate of metabolic syndrome is associated with healthcare-related costs and mortality rate [20]. The KDHP aims to develop digital health approaches to improve the personalized care of individuals with metabolic syndrome and to evaluate the benefits of individualized treatment relative to usual care. The goal of this project is to confirm the usefulness and reliability of personalized health management systems by reinforcing lifestyle changes. In the present study, in addition to a feedback advice system, a smartphone app (CARADA) was used to collect data on activity, food or water intake, and sleep. The feasibility of a mobile digital intervention depends on personal literacy [21, 22]. Therefore, we examined the change in night-time frequency and HUS over 6 months using variables as covariates: age, sex, and education (high school graduate or less, and above high school graduate). The reliability of this mobile digital intervention was validated, as night-time frequency worsened in the non-intervention group over 6 months of 24-h voided volume monitoring using s-HMSU. However, night-time frequency did not change in the intervention group over 6 months. Thus, long-term observation might be needed to confirm the effect of an intervention. In the present study, parameters related to metabolic syndrome such as body weight, abdominal circumference, blood pressure, and HbA1c did not change in either group. Recently, a 4-year cohort study involving 5234 Japanese individuals (1173 men and 4061 women; mean age, 55.6 years) had revealed the association between nocturia and the incidence of metabolic syndrome (odds ratio, 2.90) [23]. The results of our interventional study and this previous cohort study indicate that nocturia could be a symptom of metabolic syndrome and may appear or be improved in the early phase of metabolic syndrome onset or improvement. The autonomic spinobulbospinal reflex pathway [24] and other visceral organs regulate voiding; thus, voiding-related parameters might be sensitive to physical changes. Further studies are necessary to clarify this point. Now that we have constructed a feedback advice system including daily activity from the KDHP data, the next step will be to create a novel advice system that combines daily activity and voiding information.

This study has several limitations that should be acknowledged. First, the majority of participants could only begin to use 24-h voided volume monitoring halfway of total intervention period 15 months after it was fixed at each home toilet. The mean follow-up period was 6.7 months, and the complete rate for 6 months was 72%. In addition, random allocation for comparison was not guaranteed for the intervention and non-intervention groups, due to the selection flow (Fig. 1), although the comparison was made after allowing for age, sex, and education. Therefore, caution was exercised when interpreting our results. Second, it was insufficient to perform 24-h monitoring for just one day every 3 months for a follow-up period of 6 months to clarify the improvement of nocturia. Third, our study participants comprised middle-aged people (mean age 51.8 years) who had relatively mild nocturia, as well as metabolic disorders. The effect of our mobile digital intervention on nocturia may be different from that we witnessed in this study, if those with moderate-to-severe nocturia are targeted. Fourth, we did not evaluate the relationship between improvement in metabolic disorders and change in night-time frequency according to the personal literacy, such as the frequency of data entry. Despite these limitations, we believe that a mobile digital intervention in combination with a 24-h voided volume monitoring system is useful to reinforce lifestyle modification in individuals with metabolic disorders and to improve nocturia.

## Conclusions

The s-HMSU provides a reliable measurement of voiding information for home use. Voiding information obtained from this system, such as night-time frequency and nocturnal polyuria index, might be a surrogate marker of metabolic syndrome, which can reinforce lifestyle changes in individuals with metabolic disorders.

## Data Availability

The datasets analyzed during the current study are available from the corresponding author on reasonable request.
